# Supplementary material for: Disparities in prostate cancer screening practices among general practitioners and urologists (PROSHADE study): A cross-sectional study
Source: PLoS One. 2026 May 21;21(5):e0349758. doi: 10.1371/journal.pone.0349758 (PMC13193549; doi:10.1371/journal.pone.0349758)
Supplement: S2 — (DOCX) [file pone.0349758.s002.docx]

# QUESTIONNAIRE

# A. IDENTIFICATION DATA

Sex:

Age:

Specialty:

Years in practice (including specialty):

Type of institution where you work (public, private, or both):

Level of care where you work (emergency, primary care, or specialties):

# B. TRAINING ON PSA

**B.1. Have you received specific training aimed at understanding the indications of PSA testing and its clinical management?**

Yes ( )  No ( )

If yes, please specify the context: During undergraduate/graduate training, During specialty training, In professional practice.

Do you consider that you have sufficient information about PSA and its appropriate use?

Yes ( )  No ( )

# C. USE OF PSA TESTING

**C1. At what age would you request the first PSA test in an asymptomatic man?**

40–49 ( ) 50–59 ( ) Over 60 ( ) Would not request ( )

**C2. In which age decade do you request the greatest number of PSA tests?**

40–49 ( ) 50–59 ( ) 60–69 ( ) 70–79 ( )

**C3. In which age decade do you think PSA has the highest sensitivity for diagnosing prostate cancer?**

40–49 ( ) 50–59 ( ) 60–69 ( ) 70–79 ( ) N/A ( )

**C4. At what age do you think PSA testing should be discontinued in men with normal values?**

70 ( ) 75 ( ) 80 ( ) Never ( ) None of the above ( )

**C5. How often would you approximately request PSA testing in a man with previously normal levels and within the appropriate age range?**

Every <6 months ( ) Every 6 months ( ) Every 9 months ( ) Every year ( )

Every 2 years ( ) Every >2 years ( )

**C6. If a patient actively requests the PSA test, I…**

( ) Request it without explanation.

( ) Explain the (dis)advantages of the test and, if indicated, request it.

( ) Explain the (dis)advantages of the test and request it even if not indicated.

Other: ____________________________________

**C7. How many PSA determinations per year would you request in a 65-year-old man with no treatment and a last PSA of 3 ng/ml one year ago?**

None ( ) One ( ) Two ( ) Three ( )

Do you offer PSA testing to men undergoing blood tests for other reasons? Do you offer PSA testing to men attending for another reason and for whom you plan to perform blood tests?

Yes ( ) No ( )

If you have decided PSA is indicated for a patient:

( ) I include it in the test without asking the patient.

( ) I ask the patient whether they wish to do it and explain pros and cons.

( ) I provide a standardized written form.

Other: ________________________________

**C8. When referring a patient for PSA testing, do you inform them about the following potential advantages and disadvantages of screening?**

|  | **Never** | **Almost never** | **Sometimes** | **Often** | **Always** |
| --- | --- | --- | --- | --- | --- |
| Impact on overall mortality | ☐ | ☐ | ☐ | ☐ | ☐ |
| Impact on disease-specific mortality | ☐ | ☐ | ☐ | ☐ | ☐ |
| Impact on metastasis likelihood | ☐ | ☐ | ☐ | ☐ | ☐ |
| Early cancer detection | ☐ | ☐ | ☐ | ☐ | ☐ |
| Opportunity for less aggressive treatments | ☐ | ☐ | ☐ | ☐ | ☐ |
| Inform about other relevant aspects | ☐ | ☐ | ☐ | ☐ | ☐ |

|  | **Never** | **Almost never** | **Sometimes** | **Often** | **Always** |
| --- | --- | --- | --- | --- | --- |
| Overdiagnosis | ☐ | ☐ | ☐ | ☐ | ☐ |
| Possibility of false positives | ☐ | ☐ | ☐ | ☐ | ☐ |
| Anxiety while waiting for results | ☐ | ☐ | ☐ | ☐ | ☐ |
| Need for additional diagnostic tests if PSA is elevated | ☐ | ☐ | ☐ | ☐ | ☐ |
| Consequences of medical treatment | ☐ | ☐ | ☐ | ☐ | ☐ |
| Inform about other relevant aspects | ☐ | ☐ | ☐ | ☐ | ☐ |

**C9. Do you make the decision to request PSA testing jointly with the patient?**

Yes ( ) No ( )

# D. GUIDELINES AND RECOMMENDATIONS

**D1. Are you familiar with the following guidelines or recommendations on early detection of prostate cancer?**

|  | I know the content | I’ve read it but don’t know the content in detail | I’ve heard of it | I don’t know it | |
| --- | --- | --- | --- | --- | --- |
| European guideline on prostate cancer (European Association of Urology), Spanish Association of Urology | ☐ | ☐ | ☐ | | ☐ |
| US Preventive Services Task Force (USPSTF) | ☐ | ☐ | ☐ | | ☐ |
| Preventive Activities and Health Promotion Program of the Spanish Society of Family and Community Medicine | ☐ | ☐ | ☐ | | ☐ |
| Other guidelines (specify): ________________________________________________ | ☐ | ☐ | ☐ | | ☐ |

**D2. Do you use the recommendations from the European Association of Urology or others in your usual practice?**

Yes ( ) No ( )

**D3. Has the way you use PSA testing changed since the publication of the latest European Association of Urology recommendations?**

Yes ( ) No ( )

If yes, indicate how it has changed regarding test frequency:

Considerably less frequent / Less frequent / Same as before / More frequent / Considerably more frequent

# E. OPINIONS ON PSA

**E1. How concerned are you about missing a prostate cancer diagnosis in a patient?**

Not at all ( ) Slightly ( ) Quite concerned ( ) Very concerned ( )

**E2. How important do you think prostate cancer screening is?**

Not important ( ) Very little importance ( ) Neutral ( ) Important ( ) Very important ( )

**E4. Regarding the usefulness of PSA for prostate cancer diagnosis, you think it is:**

Not useful ( ) Slightly useful ( ) Quite useful ( ) Very useful ( )

**E5. Would you recommend PSA testing to your relatives?**

Definitely not ( ) Probably not ( ) Neutral ( ) Probably yes ( ) Definitely yes ( )
